# Supplementary material for: Proof of an Outer Membrane Target of the Efflux Inhibitor Phe-Arg-β-Naphthylamide from Random Mutagenesis
Source: Molecules. 2019 Jan 29;24(3):470. doi: 10.3390/molecules24030470 (PMC6384556; doi:10.3390/molecules24030470)
Supplement: Supplementary file 1 [file molecules-24-00470-s001.pdf]

molecules

MDPI

Supplementary Materials

Communication

Proof of an Outer Membrane Target of the Efflux Inhibitor Phe-Arg-β-naphthylamide from Random Mutagenesis

Sabine Schuster [1,*], Jürgen A. Bohnert [2], Martina Vavra [1], John W. Rossen [3] and Winfried V. Kern [1,4]

1 Division of Infectious Diseases, Department of Medicine II, University Hospital and Medical Center, Freiburg, Germany;
2 Institute of Medical Microbiology, Greifswald University Hospital, Greifswald, Germany;
3 Department of Medical Microbiology and Infection Prevention, University of Groningen, University Medical Center Groningen, Groningen, the Netherlands;
4 Faculty of Medicine, Albert-Ludwigs-University, Freiburg, Germany;
* Correspondence: sabine.schuster@uniklinik-freiburg.de; Tel.: +49-761-270-35990

Table S1 Susceptibilities of parental *E. coli* 3-AG100 and of random mutagenesis and reconstructed mutants in the absence and presence of PAβN tested in LB medium.

	MIC (mg/L) [1]											
	3-AG100 (parental *E. coli*)		Mutant C5/1/17		Mutant CP1		Mutant 3-AG00*acrB*$_{CP1}$		Mutant CP1*acrB*$_{wt}$		Mutant Δ*lpxM*	
Agent [2]	alone	with PAβN	alone	with PAβN	alone	with PAβN	alone	with PAβN	alone	with PAβN	alone	with PAβN
LVX	2	0.5	0.25	0.25	0.5	0.25	1	0.25	0.5	0.25	0.5	0.25
MXF	4	0.25	0.5	0.25	1	0.25	4	0.25	1	0.25	1	0.5
TET	4	4	4	4	2	2	2	2	4	4	4	4
MIN	4	0.25	1	0.5	2	0.5	4	0.25	2	0.5	2	0.5
OXA	512	64	128	64	128	32	256	64	256	32	256	64
LZD	1024	128	512	128	256	128	512	64	1024	256	1024	256
CHL	16	2	4	2	4	2	4	2	16	4	16	4
RIF	16	0.25	2	0.5	8	2	16	0.25	16	2	8	2
RIX	32	0.5	4	2	16	4	32	0.5	16	4	8	4
CLR	512	8	1024	1024	512	128	512	16	256	32	256	64
ERY	1024	32	1024	1024	1024	256	1024	32	512	64	1024	128
AZM	256	8	512	512	256	64	256	4	128	16	256	64
NOV	1024	8	128	8	128	8	512	4	512	64	256	8
PAβN	>512		> 512		512		>512		512		> 512	
NMP	512		512		512		512		512		512	

[1] PAβN used at 25 mg/L; [2] LVX, levofloxacin; MXV, moxifloxacin; TET, tetracycline; MIN, minocycline; OXA, oxacillin; LZD, linezolid; CHL, chloramphenicol; RIF, rifampin; RIX, rifaximine; CLR, clarithromycin; ERY, erythromycin; AZM, azithromycin; NOV, novobiocin; PAβN and NMP, efflux pump inhibitors.

Table S2 Susceptibilities of parental *E. coli* 3-AG100 and of mutant Δ*lpxM* in the absence and presence of EPIs and PMBN tested in cation-adjusted MH medium.

	**MIC (mg/L)** [1]									
	**3-AG100 (parental *E. coli*)**					**Mutant Δ*lpxM***				
**Agent** [2]	**alone**	**with PAβN**	**with NMP**	**with MBX 2319**	**with PMBN**	**alone**	**with PAβN**	**with NMP**	**with MBX 2319**	**with PMBN**
LVX	1	0.25	0.25	0.25	0.5	0.5	0.125	0.125	0.125	0.125
MXF	2	0.25	0.5	0.5	1	1	0.25	0.5	0.25	0.25
TET	4	2	1	1	3	4	2	1	0.5	1
MIN	2	0.25	0.5	1	2	2	0.5	1	1	0.5
TGC	0.75	0.38	0.25	0.5	1	0.5	0.5	0.25	0.25	0.5
OXA	1024	64	256	256	128	512	64	256	128	16
CXM	32	32	16	8	4	16	16	4	4	0.5
LZD	1024	128	128	512	256	1024	256	128	512	128
CHL	32	4	4	8	4	16	4	8	8	2
RIF	16	0.5	16	8	0.09	8	2	8	4	0.06
RIX	16	1	64	16	1.5	8	4	16	4	0.25
CLR	256	4	128	128	16	128	32	128	64	2
ERY	512	16	512	128	128	512	128	512	64	32
AZM	128	1	32	16	12	64	8	16	8	2
JOS	> 512	32	> 512	> 512	ND	512	256	512	ND	ND
NOV	> 1024	16	512	1024	48	512	32	256	256	4
VAN	512	512	512	ND	ND	128	256	256	ND	ND
GEN	1	1	1	1	1	1	1	1	1	1
PAβN	> 512					512				
NMP	512					256				
PMBN	512					64				

[1] PAβN used at 25 mg/L, NMP 100 mg/L, MBX 2319 50 μM, and PMBN 10 mg/L; ND, not determined.[2] LVX, levofloxacin; MXV, moxifloxacin; TET, tetracycline; MIN, minocycline; TGC, tigecycline; OXA, oxacillin; CXM, cefuroxime; LZD, linezolid; CHL, chloramphenicol; RIF, rifampin; RIX, rifaximine; CLR, clarithromycin; ERY, erythromycin; AZM, azithromycin; NOV, novobiocin; JOS, josamycin; GEN, gentamicin; PAβN, NMP, and MBX 2319, efflux pump inhibitors; PMBN, outer membrane permeabilizer.

Table S3. Oligonucleotides used in this study.

**Oligonucleotide**	**Sequence** (5′-3′ direction)[1]	**Application**
AcrB forward primer	agaaagtgcgtcctggtgtc	Mutazym II error prone PCR of whole *acrB* and amplification of *acrB*$_{CP1}$ and *acrB*$_{wt}$
AcrB reverse primer	gatcctgagttggtggttcaa	
Upper oligo *acrB*-(*rpsL-neo*)	tgctcagcctgaacagtccaagtcttaacttaaacaggagccgttaagacggcctggtgatgatggcgggatcg	Amplification of the *rpsL-neo* cassette for replacement of whole *acrB*
Lower oligo *acrB*-(*rpsL-neo*)	gttatgcataaaaaaggccgcttacgcggccttagtgattacacgttgtatcagaagaactcgtcaagaaggcg	
Upper oligo *lpxM*-(PGK-*gb2-neo*)	aaacttgaacttatcatcaggcgaaggcctctcctcgcgagaggcttttaattaaccctcactaaagggcg	Amplification of the PGK-*gb2-neo* cassette for insertion in *lpxM*
Lower oligo *lpxM*-(PGK-*gb2-neo*)	cgctacactatcaccagattgatttttgccttatccgaaactggaaaagctaatacgactcactatagggctc	
LpxM forward primer	gccgctacactatcaccaga	Sequencing of *lpxM*
LpxM reverse primer	tcataaatcggaaacagcggt	

[1] Underlined sequences correspond to the respective insertion cassette.

© 2018 by the authors. Submitted for possible open access publication under the terms and conditions of the Creative Commons Attribution (CC BY) license (http://creativecommons.org/licenses/by/4.0/).
